# Supplementary material for: Characteristics and Outcome of Children with Renal Cell Carcinoma: A Narrative Review
Source: Cancers (Basel). 2020 Jul 3;12(7):1776. doi: 10.3390/cancers12071776 (PMC7407101; doi:10.3390/cancers12071776)
Supplement: Supplementary file 1 [file cancers-12-01776-s001.pdf]

Supplementary Materials: Characteristics and Outcome of Children with Renal Cell Carcinoma: A Narrative Review

Justine N. van der Beek, James I. Geller, Ronald R. de Krijger, Norbert Graf, Kathy Pritchard-Jones, Jarno Drost, Arnauld C. Verschuur, Dermot Murphy, Satyajit Ray, Filippo Spreafico, Kristina Dzhuma, Annemieke S. Littooij, Barbara Selle, Godelieve A.M. Tytgat and Marry M. van den Heuvel-Eibrink

Table S1. Search strategy in Pubmed and Embase/Medline.

**Pubmed**
(((((((("Adenocarcinoma of Kidney*"[Title/Abstract]) OR "Grawitz Tumor*"[Title/Abstract]) OR "Grawitz Tumour*"[Title/Abstract]) OR "Collecting Duct Carcinoma*"[Title/Abstract]) OR Hypernephroma*[Title/Abstract]) OR "Renal-Cell Carcinoma*"[Title/Abstract]) OR "Carcinoma, Renal Cell"[Mesh])) OR (((((Carcinoma*[Title/Abstract]) OR Adenocarcinoma*[Title/Abstract]) OR Cancer*[Title/Abstract])) AND ((((Renal[Title/Abstract]) OR "Renal Cell"[Title/Abstract]) OR Nephroid[Title/Abstract]) OR Hypernephroid[Title/Abstract]))
**AND**
((((((Children[Title/Abstract]) OR Pediatric*[Title/Abstract]) OR Paediatric*[Title/Abstract]) OR "Child"[Mesh]) OR "Child, Preschool"[Mesh]) OR "Adolescent"[Mesh]) OR "Pediatrics"[Mesh]
**Embase/Medline**
(((((((((‘Adenocarcinoma of Kidney*’:ab,ti) OR ‘Grawitz Tumor*’:ab,ti) OR ‘Grawitz Tumour*’:ab,ti) OR ‘Collecting Duct Carcinoma*’:ab,ti) OR ‘Hypernephroma*’:ab,ti) OR ‘Renal-Cell Carcinoma*’:ab,ti) OR 'renal cell carcinoma'/exp)) OR (((((‘Carcinoma*’:ab,ti) OR ‘Adenocarcinoma*’:ab,ti) OR ‘Cancer*’:ab,ti)) AND ((((‘Renal’:ab,ti) OR ‘Renal Cell’:ab,ti) OR ‘Nephroid’:ab,ti) OR ‘Hypernephroid’:ab,ti)))
**AND**
(((((((‘Child’:ab,ti) OR ‘Children’:ab,ti) OR ‘Pediatric*’:ab,ti) OR ‘Paediatric*’:ab,ti) OR 'child'/exp) OR ‘adolescent’/exp))

* searched for singular and pleural terms.

Table S2. Transparency regarding patients in the articles identified after title and abstract screening using the in- and exclusion criteria.

**Author (Year)**	**Year(s) of Inclusion**	**Demographic Information (Registry and/or Country and/or City)**	**Included (IN)/Excluded (EX)**
		**National Cancer Database (NCD/NCDB)**	
Akhavan (2015) [1]	1998–2011	NCDB (<30 years)	EX
Rialon (2015) [2]	1998–2011	NCDB (0–17 years)	EX
		**AREN03B2**	
Cajaiba (2018) [3]	August 2006–July 2016	AREN03B2	IN
Geller (2015) [4]	Unknown	AREN03B2	EX
		**United States of America**	
Ambalavanan (2019) [5]	2000–2015	Cincinnati Children’s Hospital and Medical Center (Cincinnati)	EX
Wang (2012) [6]	1995–2011	2 academic institutions (Atlanta / Indianapolis)	IN
Geller (2008) [7]	Unknown	Cincinnati Children’s Hospital and Medical Center (Cincinnati)	IN
Silberstein (2009) [8]	1988–2004	California cancer registry	IN
Wu (2008) [9]	January 1986–June 2006	Michigan School of Medicine	IN

Estrada (2005) [10]	1965–2003	Children's Hospital Boston (Boston)	IN
Geller (2004) [11]	1962–Unknown	St. Jude Children's Research Hospital (Memphis)	IN
Aronson (1996) [12]	May 1956–July 1990	Memorial Sloan Kettering (New York)	IN
Dehner (1970) [13]	Unknown	Kidney tumor registry and registry of pediatric pathology of the American registry of Pathology (Washington DC)	IN
		**Canada**	
Ramphal (2006) [14]	January 1984–December 2003	The Hospital for Sick Children (Toronto)	IN
Cook (2006) [15]	1980–2005	The Hospital for Sick Children (Toronto)	EX
Carcao (1998) [16]	1979–1996	The Hospital for Sick Children (Toronto)<br>Montreals Children's Hospital (Montreal)<br>Hospital st. Justine (Montreal)	EX
Chan (1983) [17]	1958–1982	The Hospital for Sick Children (Toronto)<br>Princess Margaret Hospital (Toronto)	IN
		**Europe**	
Indolfi (2012) [18]	January 1973–November 2010	AIEOP	EX
Varan (2007) [19]	1972–2004	Single center study (Ankara, Turkey)	IN
Selle (2006) [20]	1980–2005	GCCR & KTR (Germany)	IN
Indolfi (2003) [21]	January 1973–January 2001	AIEOP	IN
		**China/South Korea**	
Kim (2015) [22]	1988–2014	Dongnam Institute of Radiologial & Medical Sciences Cancer center (Busan)<br>Samsung Medical Center (Seoul)<br>Asan Medical Center (Seoul)<br>Seoul National University Hospital (Seoul)<br>Seoul St. Mary's Hospital (Seoul)	IN
Rao (2011) [23]	1989–2008	Nanjing Jinling Hospital (Nanjing)<br>Nanjing Drum Tower Hospital (Nanjing)	IN
Baek (2010) [24]	1997–2008	Samsung Medical Center<br>Masan Samsung Hospital (Seoul/Masan)	EX

Table S3. The Modified Robson Staging System and TNM-Staging Systems of RCC. (**a**) Stage grouping of the Modified Robson Staging System and the TNM-Staging Systems of RCC [25–29]; (**b**). TMN-Staging Systems of RCC [25–29].

**a**	
**Modified Robson Staging System of RCC**	
Stage I	Limited to the kidney
Stage II	Involvement of perinephric fat or adrenal gland, but remains limited to Gerota fascia
Stage IIIa	Renal vein or caval involvement below and above the diaphragm
Stage IIIb	Lymph node involvement
Stage IIIc	Both IIIa and IIIb: combination of venous and nodal involvement
Stage IVa	Direct invasion of adjacent organs / structures: local extension beyond Gerota fascia

Stage IVb	Distant metastases		
	**1997 TNM Staging System of RCC**		
Stage I	T1	N0	M0
Stage II	T2	N0	M0
Stage III	T1	N1	M0
	T2	N1	M0
	T3	N0, N1	M0
Stage IV	T4	N0, N1	M0
	Any T	N2	M0
	Any T	Any N	M1
	**2002 TNM Staging System of RCC**		
Stage I	T1	N0	M0
Stage II	T2	N0	M0
Stage III	T1	N1	M0
	T2	N1	M0
	T3	N0, N1	M0
Stage IV	T4	Any N	M0
	Any T	N2	M0
	Any T	Any N	M1
	**2010 TNM Staging System of RCC**		
Stage I	T1	N0	M0
Stage II	T2	N0	M0
Stage III	T1	N1	M0
	T2	N1	M0
	T3	N0, N1	M0
Stage IV	T4	Any N	M0
	Any T	Any N	M1

b

Year	**1997**	**2002**	**2010**
		**T–primary tumour**	
Tx	Cannot be assessed	Cannot be assessed	Cannot be assessed
T0	No evidence	No evidence	No evidence
T1	≤7.0 cm, limited to kidney	≤7.0 cm, limited to kidney	≤7.0 cm, limited to kidney
T1a	-	≤4.0 cm, limited to kidney	≤4.0 cm in greatest dimension, limited to kidney
T1b	-	4–7 cm, limited to kidney	4–7 cm, limited to kidney
T2	>7.0 cm, limited to kidney	>7.0 cm, limited to kidney	>7.0 cm, limited to kidney
T2a	-	-	>7.0 cm, but ≤10 cm, limited to kidney
T2b	-	-	>10cm, limited to the kidney
T3	Extends into major veins or invades adrenal gland or perinephric tissues but not beyond Gerota fascia	Extends into major veins or invades adrenal glad or perinephric fat but not beyond Gerota fascia	Extends into major veins or perinephric tissues but not into the ipsilateral adrenal gland and not beyond Gerota fascia
T3a	Invades adrenal gland or perinephric tissues, but not beyond Gerota fascia	Invades adrenal gland or perinephral and/or renal sinus fat but not beyond Gerota fascia	Grossly extends into the renal vein or its segmental (muscle containing) branches, or invades perirenal

			and/or renal sinus fat but not beyond Gerota fascia
T3b	Grossly extends into renal vein(s) or vena cava below diaphragm	Extends into renal vein or its segmental branches or infradiaphragmatic vena cava	Grossly extends into the vena cava below the diaphragm
T3c	Grossly extends into vena cava above diaphragm	Extends into superdiaphragmatic vena cava or invades wall of vena cava	Grossly extends into the vena cava above the diaphragm or invades wall of the vena cava
T4	Invades beyond Gerota fascia	Invades beyond Gerota fascia	Invades beyond Gerota fascia (including contiguous extension into the ipsilateral adrenal gland)
		**N-regional lymph nodes**	
Nx	Cannot be assessed	Cannot be assessed	Cannot be assessed
N0	No metastasis	No metastasis	No metastasis
N1	Metastasis in single regional lymph node	Metastasis in single regional lymph node	Metastases in regional lymph node(s)
N2	Metastasis in >1 regional lymph node	Metastasis in >1 regional lymph node	-
		**M–Distant Metastasis**	
Mx	Cannot be assessed	Cannot be assessed	Cannot be assessed
M0	Absent	Absent	Absent
M1	Present	Present	Present

Table S4. Studies based on multi-center registration and/or databases.

Author (Year)	Amount of Patients	Country	Registry	Years of Inclusion
Cajaiba (2018) [3]	208	USA	AREN03B2	August 2006–July 2016
Akhavan (2015) [1]	515	USA	NCDB	1998–2011
Geller (2015) [4]	120	USA	AREN03B2	*NS, <2015*
Rialon (2015) [2]	304	USA	NCDB	1998–2011
Indolfi (2012) [18]	14	Italy	AIEOP	January 1973–November 2010
Silberstein (2009) [8]	43	USA	California Cancer Registry	1988–2004
Selle (2006) [20]	49	Germany	GCCR & KTR	1980–2005
Indolfi (2003) [21]	41	Italy	AIEOP	January 1973–January 2001
Dehner (1970) [13]	14	USA	American registry of pathology	NS, <1970

The other studies were mainly single-center studies. USA = United States of America; AREN03B2 = The Children's Oncology Group (COG) renal tumor study; NCDB = National Cancer Database; AIEOP = Italian Association for Pediatric Hematology and Oncology; GCCR = German Childhood Cancer Registry; KTR = Kiel Paediatric Tumour Registry.

Table S5. Available included studies with information about survival according to tumor stage.

Author (Year)	Nr.[ϒ]	Number of Patients	Staging System	Stage I		Stage II		Stage III		Stage IV	
				Nr. of Patients	Survival % (Range)	Nr. of Patients	Survival % (Range)	Nr. of Patients	Survival % (Range)	Nr. of Patients	Survival % (Range)
Silberstein (2009) [8]	5	43	TNM	23	88.5% (SE7.7%)			9	58.1% (SE 18.9%)	11	9.1% (SE 8.0%)
Selle (2006) [20]	9	49	TNM	28	EFS: 96.4%			8 (N1/2+M0)<br>4 (M1)	EFS: 68.8%<br>EFS: 25.0%		
Indolfi (2003) [21]	13	41	Modified Robson	19	20yEFS: 88.9% (95%CI 163–100%)<br>20yOS: 88.9% (95%CI 163–100%)			21	20yEFS: 18% (95%CI 10–36%) $p = 0.0001$<br>20yOS: 22.6% (95%CI 12–43%) $p = 0.0001$		
Geller (2004) [11] Systematic review	12	-	Modified Robson	243	92%	26	85%	66	73%	72	14%

ϒ Article number referring to Table 1. 95%CI = 95% confidence interval; 1yS = one-year survival; 5yS = five-year survival; SE = standard error; EFS = event-free survival; OS = overall survival; y = year; m = month; NS = not specified

Table S6. Genes and syndromes associated with RCC [9,20,23,30–33].

Genes	Associated Syndromes
VHL	Von Hippel-Lindau syndrome
TSC1	Tuberous sclerosis
TSC2	
TP53	Li-Fraumeni syndrome
FLCN	Birt-Hogg-Dubé syndrome
FH	Hereditary leiomyomatosis and renal cell cancer
PTEN	PTEN Hamartoma Tumor Syndrome / Cowden syndrome
SDHA	Hereditary paraganglioma-pheochromocytoma syndromes
SDHB	
SDHC	
SDHD	
TMEM127	
MLH1	Lynch syndrome
MSH2	
MSH6	
PMS2	
EPCAM	
BAP1	-
MET	Herediatry papillary renal cancer
CDKN2B	-

References

1. Akhavan, A.; Richards, M.; Shnorhavorian, M.; Goldin, A.; Gow, K.; Merguerian, P.A. Renal cell carcinoma in children, adolescents and young adults: A National Cancer Database study. *J. Urol.* 2015, *193*, 1336–1341, doi:10.1016/j.juro.2014.10.108.
2. Rialon, K.L.; Gulack, B.C.; Englum, B.R.; Routh, J.C.; Rice, H.E. Factors impacting survival in children with renal cell carcinoma. *J. Pediatr. Surg.* **2015**, *50*, 1014–1018, doi:10.1016/j.jpedsurg.2015.03.027.
3. Cajaiba, M.M.; Dyer, L.M.; Geller, J.I.; Jennings, L.J.; George, D.; Kirschmann, D.; Rohan, S.M.; Cost, N.G.; Khanna, G.; Mullen, E.A., et al. The classification of pediatric and young adult renal cell carcinoma registered on the children's oncology group (COG) protocol AREN03B2 after focused genetic testing. *Cancer* **2018**, *124*, 3381–3389, doi:10.1002/cncr.31578.
4. Geller, J.I.; Ehrlich, P.F.; Cost, N.G.; Khanna, G.; Mullen, E.A.; Gratias, E.J.; Naranjo, A.; Dome, J.S.; Perlman, E.J. Characterization of adolescent and pediatric renal cell carcinoma: A report from the Children's Oncology Group study AREN03B2. *Cancer* **2015**, *121*, 2457–2464, doi:10.1002/cncr.29368.
5. Ambalavanan, M.; Geller, J.I. Treatment of advanced pediatric renal cell carcinoma. *Pediatr. Blood Cancer* 2019, *66*, e27766, doi:10.1002/pbc.27766.
6. Wang, J.; Shehata, B.M.; Langness, S.M.; Davis, G.K.; Cheng, L.; Osunkoya, A.O. Clear cell, papillary and chromophobe renal cell carcinoma in patients younger than 20 years old: A clinicopathologic study with follow-up. *J. Pediatr. Urol.* **2012**, *8*, 531–534, doi:10.1016/j.jpurol.2011.09.010.
7. Geller, J.I.; Argani, P.; Adeniran, A.; Hampton, E.; De Marzo, A.; Hicks, J.; Collins, M.H. Translocation renal cell carcinoma: Lack of negative impact due to lymph node spread. *Cancer* **2008**, *112*, 1607–1616, doi:10.1002/cncr.23331.
8. Silberstein, J.; Grabowski, J.; Saltzstein, S.L.; Kane, C.J. Renal cell carcinoma in the pediatric population: Results from the California Cancer Registry. *Pediatr. Blood Cancer* **2009**, *52*, 237–241, doi:10.1002/pbc.21779.
9. Wu, A.; Kunju, L.P.; Cheng, L.; Shah, R.B. Renal cell carcinoma in children and young adults: Analysis of clinicopathological, immunohistochemical and molecular characteristics with an emphasis on the spectrum of Xp11.2 translocation-associated and unusual clear cell subtypes. *Histopathology* **2008**, *53*, 533–544, doi:10.1111/j.1365-2559.2008.03151.x.
10. Estrada, C.R.; Suthar, A.M.; Eaton, S.H.; Cilento, B.G., Jr. Renal cell carcinoma: Children's Hospital Boston experience. *Urology* **2005**, *66*, 1296–1300, doi:10.1016/j.urology.2005.06.104.

11. Geller, J.I.; Dome, J.S. Local lymph node involvement does not predict poor outcome in pediatric renal cell carcinoma. *Cancer* **2004**, *101*, 1575–1583, doi:10.1002/cncr.20548.
12. Aronson, D.C.; Medary, I.; Finlay, J.L.; Herr, H.W.; Exelby, P.R.; La Quaglia, M.P. Renal cell carcinoma in childhood and adolescence: A retrospective survey for prognostic factors in 22 cases. *J. Pediatr. Surg.* **1996**, *31*, 183–186, doi:10.1016/s0022-3468(96)90344-9.
13. Dehner, L.P.; Leestma, J.E.; Price, E.B., Jr. Renal cell carcinoma in children: A clinicopathologic study of 15 cases and review of the literature. *J. Pediatr.* **1970**, *76*, 358–368.
14. Ramphal, R.; Pappo, A.; Zielenska, M.; Grant, R.; Ngan, B.Y. Pediatric renal cell carcinoma: Clinical, pathologic, and molecular abnormalities associated with the members of the mit transcription factor family. *Am. J. Clin. Pathol.* **2006**, *126*, 349–364, doi:10.1309/98ye9e442ar7lx2x.
15. Cook, A.; Lorenzo, A.J.; Salle, J.L.; Bakhshi, M.; Cartwright, L.M.; Bagi, D.; Farhat, W.; Khoury, A. Pediatric renal cell carcinoma: Single institution 25-year case series and initial experience with partial nephrectomy. *J. Urol.* **2006**, *175*, 1456–1460, doi:10.1016/s0022-5347(05)00671-3.
16. Carcao, M.D.; Taylor, G.P.; Greenberg, M.L.; Bernstein, M.L.; Champagne, M.; Hershon, L.; Baruchel, S. Renal-cell carcinoma in children: A different disorder from its adult counterpart? *Med. Pediatr. Oncol.* **1998**, *31*, 153–158.
17. Chan, H.S.; Daneman, A.; Gribbin, M.; Martin, D.J. Renal cell carcinoma in the first two decades of life. *Pediatr. Radiol.* **1983**, *13*, 324–328, doi:10.1007/BF01625958.
18. Indolfi, P.; Spreafico, F.; Collini, P.; Cecchetto, G.; Casale, F.; Terenziani, M.; Schiavetti, A.; Pierani, P.; Piva, L.; Cuzzubbo, D., et al. Metastatic renal cell carcinoma in children and adolescents: A 30-year unsuccessful story. *J. Pediatr Hematol. Oncol.* **2012**, *34*, 277–281, doi:10.1097/MPH.0b013e318267fb12.
19. Varan, A.; Akyuz, C.; Sari, N.; Buyukpamukcu, N.; Caglar, M.; Buyukpamukcu, M. Renal cell carcinoma in children: Experience of a single center. *Nephron. Clin. Pract.* **2007**, *105*, 58–61, doi:10.1159/000097599.
20. Selle, B.; Furtwangler, R.; Graf, N.; Kaatsch, P.; Bruder, E.; Leuschner, I. Population-based study of renal cell carcinoma in children in Germany, 1980-2005: More frequently localized tumors and underlyin disorders compared with adult counterparts. *Cancer* **2006**, *107*, 2906–2914, doi:10.1002/cncr.22346.
21. Indolfi, P.; Terenziani, M.; Casale, F.; Carli, M.; Bisogno, G.; Schiavetti, A.; Mancini, A.; Rondelli, R.; Pession, A.; Jenkner, A., et al. Renal cell carcinoma in children: A clinicopathologic study. *J. Clin. Oncol.* **2003**, *21*, 530–535, doi:10.1200/jco.2003.02.072.
22. Kim, J.H.; Seo, S.I.; Song, C.; Chung, J.; Kwak, C.; Hong, S.H. Clinicohistological characteristics of renal cell carcinoma in children: A multicentre study. *Can. Urol. Assoc. J.* **2015**, *9*, 705–708, doi:10.5489/cuaj.2855.
23. Rao, Q.; Chen, J.Y.; Wang, J.D.; Ma, H.H.; Zhou, H.B.; Lu, Z.F.; Zhou, X.J. Renal cell carcinoma in children and young adults: Clinicopathological, immunohistochemical, and VHL gene analysis of 46 cases with follow-up. *Int. J. Surg. Pathol.* **2011**, *19*, 170–179, doi:10.1177/1066896909354337.
24. Baek, M.; Jung, J.Y.; Kim, J.J.; Park, K.H.; Ryu, D.S. Characteristics and clinical outcomes of renal cell carcinoma in children: A single center experience. *Int. J. Urol.* **2010**, *17*, 737–740, doi:10.1111/j.1442-2042.2010.02588.x.
25. Compton CC, B.D., Garcia-Aguilar J, Kurtzman SH, Olawaiye A, Washington MK. *AJCC Cancer Staging Atlas*; Springer: Switzerland, 2012.
26. Edge, S.B.; Compton, C.C. The American Joint Committee on Cancer: The 7th edition of the AJCC cancer staging manual and the future of TNM. *Ann. Surg. Oncol.* **2010**, *17*, 1471–1474, doi:10.1245/s10434-010-0985-4.
27. Robson, C.J.; Churchill, B.M.; Anderson, W. The results of radical nephrectomy for renal cell carcinoma. *J. Urol.* **1969**, *101*, 297-301, doi:10.1016/j.juro.2016.10.095.
28. Guinan, P.; Sobin, L.H.; Algaba, F.; Badellino, F.; Kameyama, S.; MacLennan, G.; Novick, A. TNM staging of renal cell carcinoma: Workgroup No. 3. Union International Contre le Cancer (UICC) and the American Joint Committee on Cancer (AJCC). *Cancer* **1997**, *80*, 992–993.
29. Greene, F.L.; P.D., Fleming, I.D.; et al, eds. AJCC Cancer Staging Manual. 6th ed. Springer: New York, NY, USA, 2002.
30. Shuch, B.; Vourganti, S.; Ricketts, C.J.; Middleton, L.; Peterson, J.; Merino, M.J.; Metwalli, A.R.; Srinivasan, R.; Linehan, W.M. Defining early-onset kidney cancer: Implications for germline and somatic mutation testing and clinical management. *J. Clin. Oncol.* **2014**, *32*, 431–437, doi:10.1200/jco.2013.50.8192.
31. Haas, N.B.; Nathanson, K.L. Hereditary kidney cancer syndromes. *Adv. Chronic. Kidney Dis.* **2014**, *21*, 81–90, doi:10.1053/j.ackd.2013.10.001.

32. Hol, J.A.; Jongmans, M.C.J.; Littooij, A.S.; de Krijger, R.R.; Kuiper, R.P.; van Harssel, J.J.T.; Mensenkamp, A.; Simons, M.; Tytgat, G.A.M.; van den Heuvel-Eibrink, M.M., et al. Renal cell carcinoma in young FH mutation carriers: Case series and review of the literature. *Fam Cancer* **2020**, *19*, 55–63, doi:10.1007/s10689-019-00155-3.
33. Carlo, M.I.; Mukherjee, S.; Mandelker, D.; Vijai, J.; Kemel, Y.; Zhang, L.; Knezevic, A.; Patil, S.; Ceyhan-Birsoy, O.; Huang, K.C., et al. Prevalence of germline mutations in cancer susceptibility genes in patients with advanced renal cell carcinoma. *JAMA Oncol.* **2018**, *4*, 1228–1235, doi:10.1001/jamaoncol.2018.1986.

© 2020 by the authors. Licensee MDPI, Basel, Switzerland. This article is an open access article distributed under the terms and conditions of the Creative Commons Attribution (CC BY) license (http://creativecommons.org/licenses/by/4.0/).
